# Temperature and Infill Density Effects on Thermal, Mechanical and Shape Memory Properties of Polylactic Acid/Poly(ε-caprolactone) Blends for 4D Printing

**DOI:** 10.3390/ma15248838

**Published:** 2022-12-10

**Authors:** Ang Li, Xin-Gang Chen, Lan-Ying Zhang, Yang-Fei Zhang

**Affiliations:** School of Materials Science and Engineering, Peking University, Beijing 100871, China

**Keywords:** shape memory, 4D printing, polylactic acid, poly(ε-caprolactone), model

## Abstract

Polylactic acid (PLA)/poly(ε-caprolactone) (PCL) blends have exhibited good shape memory properties and degradable characteristics in various 4D printing fields such as biomedicine, flexible electronics, and soft robotics, where the service temperature fluctuates easily by environment temperature and polymer characteristics. In this work, printed PLA/PCL 4D samples with different infill densities were prepared by material extrusion printing of pre-extruded filaments and characterized under different temperatures. The results show that the microstructures of printed samples are not influenced by printing process and have similar unique orientation as that of filaments. The thermal properties are stable and show obvious phase transition temperatures, while the mechanical properties decrease slightly in low temperature region and then decrease rapidly when temperature is over 60 °C. The increase in infill density can further improve the storage modulus more than 40% and have no significant influence on the thermal properties. The printed samples also exhibit good shape memory performances with fast recovery speeds less than 22 s. Furthermore, a two-step model is provided to predict the effective modulus of printed PLA/PCL samples and agrees well with experimental data. The results prove that temperature and infill density have different influences on the thermal, mechanical and shape memory properties of PLA/PCL blends.

## 1. Introduction

Three-dimensional (3D) printing, also known as additive manufacturing, is a rapidly developed material manufacturing method to produce complex and customized structures through layer-by-layer printing [1,2]. 4D printing is the upgrade technology of 3D printing and adds time as the fourth dimension for automatic deformation and assembling of structures under external driven factors [3]. Shape memory polymers (SMP) are widely used programmable materials for 4D printing with advantages of low cost, simple manufacture and complex structures, reverting from temporary shape to permanent shape under specific driven factor of heat, light, electricity, magnetic or laser [4,5,6,7]. The emergence of 4D printing has also brought a new direction for the preparation and application of SMP. Ge et al. [8] have reported a 4D printing approach based on high resolution projection micro-stereo-lithography and created a high resolution SMP architecture of Eiffel Tower. Zhang et al. [9] have presented a paradigm to design and manufacture a fast response gripper by embedding a SMP layer into 3D printed soft actuator. Melocchi et al. [10] have reported a printed helix SMP structure of stomach capsules, which can expand to the original shape in aqueous fluids at 37 °C.

For thermal driven SMP, after several external forces and temperature changes in shape memory process, the movement of SMP molecular chain is locked and the shape of SMP is fixed to the temporary shape [11,12]. As the temporary shape is more unstable than the permanent shape, the internal stress and energy of SMP will be released when heated to the glass transition temperature or melting temperature, while the molecular chain regains its mobility and the SMP return its permanent shape [13]. The shape memory effect has been found in a variety of polymers, which have been used in many applications including aerospace engineering [14,15], biomedicine [16,17], flexible electronics [18,19], soft robotics [20,21], etc.

Thermoplastic polymers are ideal materials for 4D printing due to their low thermal expansion coefficient, glass transition temperature and melting temperature [22]. Polylactic acid (PLA) and poly(ε-caprolactone) (PCL) are considered as the optimal candidates for 4D printing because of their good shape memory and biodegradable properties and have great potential in manufacturing of variety smart structures [23,24]. In recent years, many works have been studies on the preparation, characterization and mechanisms of PLA/PCL blends [25,26,27]. Ma et al. [28] have fabricated PLA/PCL shape memory blends for 4D printing technology and found that the blends have fast shape fixation/recovery rates with recovery time less than 1.2 s and lower glass transition temperature with the increase in PCL content. Wang et al. [29] have printed PLA/PCL blends with the composition ratios of PCL/PLA set from 3.5/1 to 1/1 and found that PCL3/PLA1 exhibits the highest shape memory recovery ratio of 95.37%, along with programming temperature of 90 °C.

Most fabrication of 4D thermoplastic polymers structures are conducted by material extrusion (ME) method, also known as fused deposition modeling or fused filament fabrication method [30,31]. The SMP filaments are heated into semi-liquid state at the nozzle and extruded layer by layer onto the build platform where layers are fused together, and final solidify into the 4D structures [32]. Liu et al. [33] have prepared PLA/PCL blends with 10–60 wt.% of PCL for ME method and found that the shape memory performance is affected by the crystallinity of reversible phase PCL, the glass transition behavior of fixed phase PLA and the two-phase interfaces. Cheng et al. [34] have developed an UV-assisted ME technology to fabricate shape memory object of PLA/PCL copolymer with enhanced layer bond strength and good shape fixing property of fixing rate more than 97%.

The properties of printed materials and structures can be improved by modified printing parameters, such as infill density, printing pattern, orientation, angle, feeding ratio and hatch spacing gap [35,36,37,38]. Many classical methods are provided to predict the effective mechanical properties of blended polymer materials, such as the rule of mixtures (RoM), Hashin-Shtrikman bounds [39,40], Mori-Tanaka model [41], Halpin-Tsai model [42] etc. However, few theoretical models are studied to predict the properties of printed materials and improve the quality of 3D and 4D printing. Liu et al. [43] have investigated the effect of infill strategies on mechanical and shape memory properties by both experimental and theoretical methods. Yao et al. [44] have studied the effect of printing angles on ultimate tensile strength of ME printed PLA samples by classical lamination theory and verified by tensile experiments. Yang et al. [45] have investigated the effective elastic properties of printed closed-cell porous materials with different porosities by finite element method simulations and three-phase model. Gonabadi et al. [46] have proposed a numerical homogenization technique to investigate the effect of build orientation, raster angle and infill density on mechanical behaviors of printed PLA parts with cellular lattice structures.

The service temperatures of PLA/PCL blends fluctuate easily by the variation of environment temperatures and dissipative heating effect by viscoelastic damping of polymer materials under external force, deformation and vibration [47]. The mechanical properties of PLA and PCL have been found to be highly dependent on temperature with significant softening behavior when the temperature increases [48,49], which is also effective for the PLA/PCL blends [50]. In this work, PLA/PCL 4D samples with different infill densities were fabricated by ME printing of pre-extruded PLA/PCL filaments. The thermal properties mechanical properties and shape memory properties of printed PLA/PCL samples were measured and discussed according to the microstructures, as well as the influences of temperature and infill density on their properties and a two-step model for predicting effective modulus of PLA/PCL 4D samples in hexagon patterns. The study on influence mechanism and theoretical prediction model are important for the promotion and prediction of PLA/PCL blends and their composites.

## 2. Materials and Methods

### 2.1. Preparation

PLA particles were purchased from Nature Works Co. (Plymouth, MN, USA) with a melting point of 165–180 °C, a density of 1.22 g cm^−3^ and a melt flow index of 9–15 g 10 min^−1^ (210 °C, 2.16 kg). PCL particles were purchased from Perstorp Co. (Perstorp Municipality, Sweden) with a melting point of 58–60 °C, a density of 1.1 g cm^−3^ and a melt flow index of 7 g 10 min^−1^ (160 °C, 2.16 kg).

**Figure 1:**
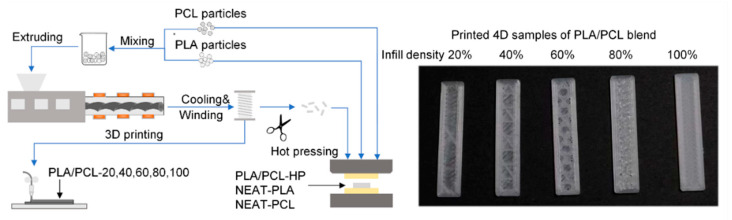
The fabrication process of PLA/PCL filaments and 4D samples by ME printing and hot pressing.

The fabrication process of PLA/PCL filaments and printed 4D samples is shown in Figure 1. PLA and PCL particles were dried at 50 °C for 24 h before preparation and then homogeneously mixed at a weight ratio of 8 : 2, which was proved to exhibit good shape memory properties [25,33]. The pre-mixed particles were poured into the cabin of a single-screw extruder (S25, Orotrim Co., Suzhou, China) at a screw speed of 25 rpm with three staged temperatures at 40 °C, 185 °C and 175 °C, which were the optimal parameters matching the physical properties of PLA/PCL [23,33] and further modified by repeated experiments with S25 extruder. The extruded blends passed through the cooling system and were collected by the coiler. Thus PLA/PCL filament with a uniform diameter of 1.75 mm was obtained.

The filament was then fed to an ME printer (Ender-3 V2, Creality Co., Shen Zhen, China), whose printing parameters had been set as in Table 1. The printed 4D samples with size of 5 mm × 30 mm × 1 mm were obtained by printing filaments with infill densities of 20%, 40%, 60%, 80% and 100% in hexagon pattern, corresponding names as PLA/PCL-20, PLA/PCL-40, PLA/PCL-60, PLA/PCL-80 and PLA/PCL-100, respectively. Additionally, neat PLA, neat PLC and blend samples with size of 5 mm × 30 mm × 1 mm were fabricated by hot-pressing of PCL particles, PCL particles and extruded PLA/PCL filaments in a PTFE mold, corresponding names as NEAT-PLA, NEAT-PCL and PLA/PCL-HP, respectively.

### 2.2. Characterization

The microstructures of PLA/PCL filaments, PLA/PCL-20/40/60/80/100 and PLA/PCL-HP samples were observed by a scanning electron microscope (SEM) (S4800, Hitachi Co., Tokyo, Japan) at an accelerating voltage of 5 kV and current of 10 μA. The surfaces of freeze-fractured samples were sputtered with a gold layer of about 10 nm. Differential scanning calorimetry (DSC) experiments were carried out using a differential scanning calorimeter (Q2000, TA Co., New Castle, DE, USA) at a heating and cooling rate of 5 °C min^−1^ from 10 °C to 190 °C in protection atmosphere of nitrogen gas. The weight loss behaviors were measured by thermogravimetric analysis (TGA) (Q600, TA Co., New Castle, DE, USA) by heating samples to 450 °C at a speed of 5 °C min^−1^. Dynamic mechanical analysis (DMA) was carried out according to the standard ASTM D5026-15, using a DMA testing machine (Q800, TA Co., New Castle, DE, USA) in film tension mode with an amplitude of 5 μm, a frequency of 1 Hz, a force track of 125%, and a heating rate of 3 °C min^−1^ during −80 °C to 120 °C.

The shape memory properties were measured by DMA in controlled force mode. The sample was heated to 65 °C and maintain 2 min with original strain recorded as $\varepsilon_{0}$. Then, a stress was gradually applied on the sample and remained constant at 0.1 MPa. In the fixing process, the temperature was gradually reduced to 20 °C and kept for 10 min to fix the temporary shape with corresponding strain recorded as $\varepsilon_{1}$. After removing the stress, the sample was kept at 20 °C for 2 min with corresponding strain recorded as $\varepsilon_{1^{\prime }}$. In the recovery process, the sample was gradually heated to 65 °C again with the stabilized strain recorded as $\varepsilon_{2}$. The fixing rate ($R_{f}$) and the recovery rate ($R_{r}$) could be calculated by the following equations [33]:

(1) $$R_{f}=\frac{\varepsilon_{1^{\prime }}-\varepsilon_{0}}{\varepsilon_{1}-\varepsilon_{0}}\times 100\%$$

(2) $$R_{r}=\frac{\varepsilon_{1}-\varepsilon_{2}}{\varepsilon_{1}-\varepsilon_{0}}\times 100\%$$

The shape memory recovery behaviors were further tested by a bending method. Printed samples with different infill densities and size of 10 mm × 60 mm × 1 mm were placed in a drying oven at 65 °C until uniformly heated. The samples were then taken out from the oven and bent at 180° with a glass rod quickly. After that, the samples were cooled to room temperature and taken back to the oven to observe the shape recovery process with a fixed camera.

## 3. Results and Discussion

### 3.1. Microstructures

**Figure 2:**
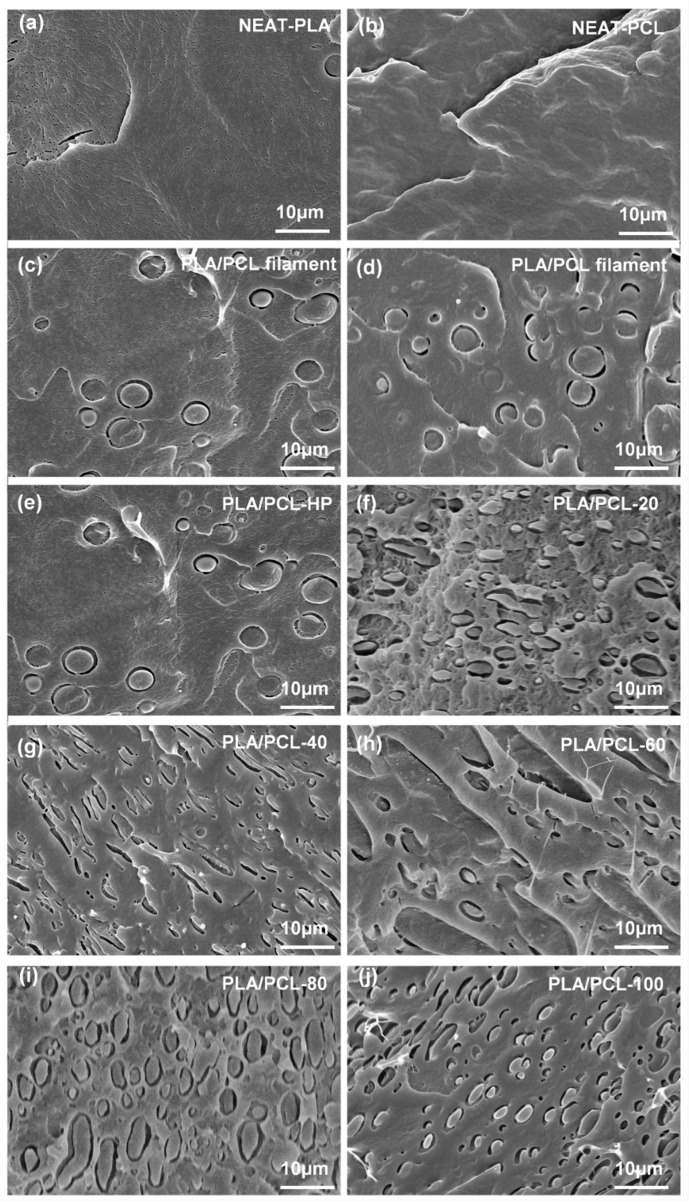
SEM images of (**a**) NEAT-PLA, (**b**) NEAT-PCL, (**c**) PLA/PCL filament along the extrude direction and (**d**) perpendicular to the extrude direction, (**e**) PLA/PCL-HP, (**f**) PLA/PCL-20, (**g**) PLA/PCL-40, (**h**) PLA/PCL-60, (**i**) PLA/PCL-80, and (**j**) PLA/PCL-100 samples.

The SEM photographs of NEAT-PCL, NEAT-PLA and extruded PLA/PCL filaments are shown in Figure 2a–d. The samples were frozen in liquid nitrogen and then brittle-fractured along different orientations. The surfaces of NEAT-PCL and NEAT-PLA are smooth and homogeneous, while the structure of NEAT-PCL is more compact than that of NEAT-PLA due to the fact that PCL is a crystalline polymer whose structure is relatively compact [27]. The fractured cross sections of PLA/PCL filaments show an obviously phase-separated microstructure as a typical sea-island structure, with spherical PCL droplets of about 10 μm homogeneous distributed in the PLA phase. There are no significant differences between the fractured cross-sections of filament along the extrude direction and perpendicular to the extrude direction.

The fractured cross-section SEM photographs of PLA/PCL filament and printed samples with different infill densities are shown in Figure 2e–j. The typical sea-island phase-separated microstructures of hot-pressed PLA/PCL blend samples show no obvious difference with that of extruded filament, while the shape of PCL droplets in the printed PLA/PCL blend samples become elliptical and partly aligned along the printing direction, forming similar anisotropy microstructures for printed samples with different infill densities. It proves that the ME printing process has significant influence on the microstructures of printed samples. The island-like PCL entirely covered by the PLA phase indicates that the movement of the PCL molecular chains are limited in this small area, which is beneficial to the shape memory process because the degree of irreversible slippage of PCL molecular chains during the shape memory cycle is much smaller than other phase structure at high content of PCL [33]. In addition, compared with NEAT-PLA, micro-pores in the PLA phase of printed sample were obviously reduced, indicating that the PLA phase has become compacter after the extrusion and printing process.

### 3.2. Thermal Properties

**Figure 3:**
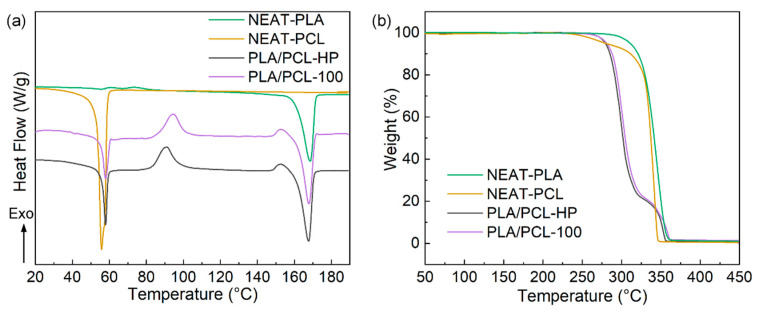
(**a**) DSC, (**b**) TGA curves of NEAT-PCL, NEAT-PLA, PLA/PCL-HP and PLA/PLC-100 samples.

The DSC and TGA curves of NEAT-PCL, NEAT-PLA, PLA/PCL-HP and PLA/PLC-100 samples are shown in Figure 3. The melting peak of NEAT-PLA is around 168 °C with melting enthalpy of approximately 39.1 J kg^−1^, while the melting peak of NEAT-PCL is around 55 °C with melting enthalpy of approximately 63.1 J kg^−1^. The DSC curves of PLA/PCL-HP and PLA/PCL-100 in Figure 3a are similar with two melting peaks around 57 °C and 168 °C, attributed to the melting behaviors of PCL and PLA phase. The exothermic peak can be obviously observed in DSC curves of NEAT-PLA, PLA/PCL-HP and PLA/PCL-100 samples, attributed to the weak mobility of PLA molecular segments in supercooled crystalline state during heating process [27]. The initial decomposition temperatures of all the samples are above 200 °C, indicating good thermal stability of hot pressed and printed samples. Comparing PLA/PCL-HP and PLA/PCL-100 samples, there is no obvious difference in their TGA curves, indicating that the fabricating method has almost no influence on the thermogravimetric property of the samples. The early decomposition of PLA/PCL blend samples is possibly attributed to the slight thermal degradation of PLA during processing and storage.

### 3.3. Mechanical Properties and Model

#### 3.3.1. Dynamic Mechanical Properties

**Figure 4:**
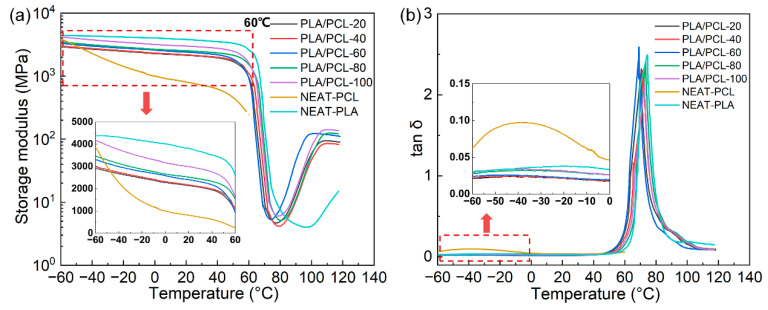
DMA curves of (**a**) storage modulus and (**b**) tan δ vs. temperatures.

Dynamic mechanical properties of NEAT-PLA, NEAT-PCL and printed samples with different infill densities are shown in Figure 4. The storage moduli of printed samples are distributed between the curves of NEAT-PLA and NEAT-PCL and improved with the increase in infill density. At 25 °C, the storage modulus of PLA/PCL-20 is 2101 MPa, while that of PLA/PCL-100 is 2954 MPa, increasing more than 40%. The storage moduli of printed samples decrease slowly in the range of −40 to 60 °C, attributed to the glass transition of PCL phase. When the temperature rises over the peak temperature of −38.06 °C as shown in the PCL tan δ curve, the storage moduli of NEAT-PCL and printed samples decrease due to the PCL phase transforming from glassy state to rubbery state. Because of PLA phase still in the glassy state, the moduli of printed samples are not significantly decreased as that of NEAT-PCL sample [51].

When the temperature rises above 60 °C, the storage moduli of printed samples decrease rapidly, mainly attributed to the glass transition of PLA phase, which can be confirmed by the tan δ curve. When the temperature rises over the peak temperature of 65 °C as shown in the PLA tan δ curve, the molecular chains in the PLA phase are released, leading to the decrease in the mechanical properties of the PLA/PCL blends [52]. Because of the cold crystallization of PLA phase, the storage moduli of printed samples increase when the temperature is above 80 °C.

#### 3.3.2. Theoretical Prediction Model

A two-step method to predict the effective modulus of printed PLA/PCL blend 4D samples under the effects of temperature and fill density is provided. As the loss modulus is small due to the value of tan δ less than 0.1, the effective modulus is approximate to the storage modulus and Young’s modulus. In this work, the Young’s modulus is approximated by the geometric average of the storage modulus and the loss modulus. The first step is prediction of the effective modulus of printed structures by Halpin-Tsai model. Considering that the microstructures of PLA/PCL blend samples in Figure 2 exhibit obvious two-phase segregated structures, where PCL droplets homogenously distributed in the PLA matrix with a clear interface between them, Halpin-Tsai model is chosen with advantages of simple form and acceptable accuracy [42]. The deformation of PCL droplets along the printing direction in the printed samples is also considered into the geometric parameters. The equations are shown below:

(3) $$\frac{P_{e}}{P_{m}}=\frac{1+\zeta \eta V}{1-\eta V}$$

(4) $$\eta =\frac{P_{f}/P_{m}-1}{P_{f}/P_{m}+\zeta }$$

where $P_{e}$ is the effective mechanical property of blend material, $P_{m}$ and $P_{f}$ are properties of matrix and the reinforcement phase, respectively. The factor ζ is used to indicate the geometry parameter of reinforcement phase, determined by the data fitting of experimental modulus curves in the specific range for various cases [53], as shown in Table 2, where E represents Young’s modulus, G represents shear modulus, and the subscripts 1 and 2 represent the length and transverse direction, respectively. In this work, the value of ζ is chosen as 3, equal to the average length-to-diameter ratio (l/d) of PCL microspheres.

The second step is prediction of the effective modulus of printed sample [54]. The 3D printed samples with different infill densities are assumed as porous materials with different porosities, for which power-law models have exhibited better results than semi-empirical models [42]. In this work, the power-law equations are chosen based on the model proposed by Gibson and Ashby [55] and modified by Blaker et al. [56], as below:

The equations are shown below:

(5) $$\frac{E}{E_{s}}=C{\left(1-\varphi \right)}^{n}$$

(6) $$\varphi =1-\frac{\rho }{\rho_{s}}$$

where φ is porosity, E and $E_{s}$ are the moduli of porous material and solid (cell wall) material with their corresponding densities of ρ and $\rho_{s}$, respectively. The constants *C* and *n* are dependent on the microstructure of the pores. According to the experimental results, the value of C can be taken as 1 [57], while the value of n is in the range of 1 to 4 [58]. Combining with these two steps, the final prediction equation for the effective modulus of printed PLA/PCL samples is given:

(7) $$E=C{\left(1-\varphi \right)}^{n}\frac{1+\zeta \frac{E_{f}/E_{m}-1}{E_{f}/E_{m}+\zeta }V}{1-\frac{E_{f}/E_{m}-1}{E_{f}/E_{m}+\zeta }V}E_{m}$$

**Figure 5:**
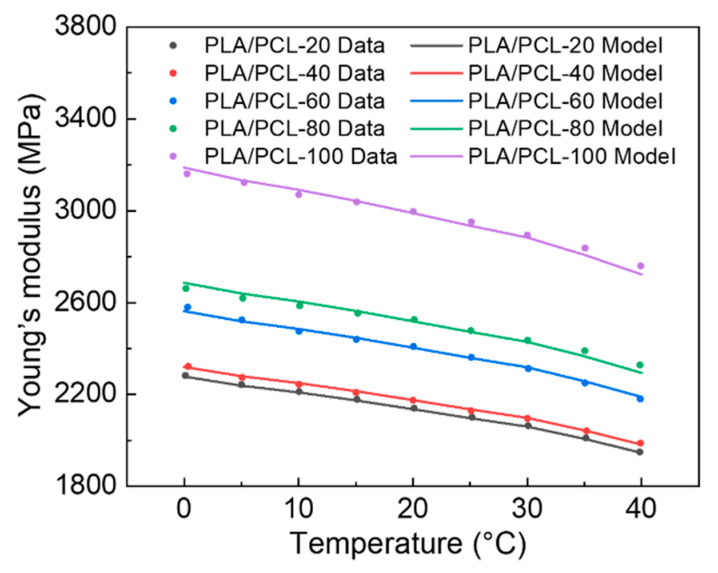
Prediction results of effective moduli versus temperatures for printed PLA/PCL samples with different infill densities.

The prediction results of effective moduli versus temperatures for printed samples with different fill densities agrees well with the experimental results, as shown in Figure 5, which proves the effectiveness of the two-step prediction model for 4D printing materials. The values of n in this two-step model are calculated by data fitting of experimental DMA curves and summarized in Table 3.

The n values for printed PLA/PCL samples with infill densities from 20% to 80% are generally distributed in the interval of 0.65–0.7, while the n value for PLA/PCL-100 is much smaller, which is attributed to the print pattern and porosity. When the infill density rises to 100%, PLA/PCL-100 cannot be assumed as a porous material, whose print pattern is corresponding changed from hexagon pattern to a solid consisted by lines [59].

### 3.4. Shape Memory Properties

**Figure 6:**
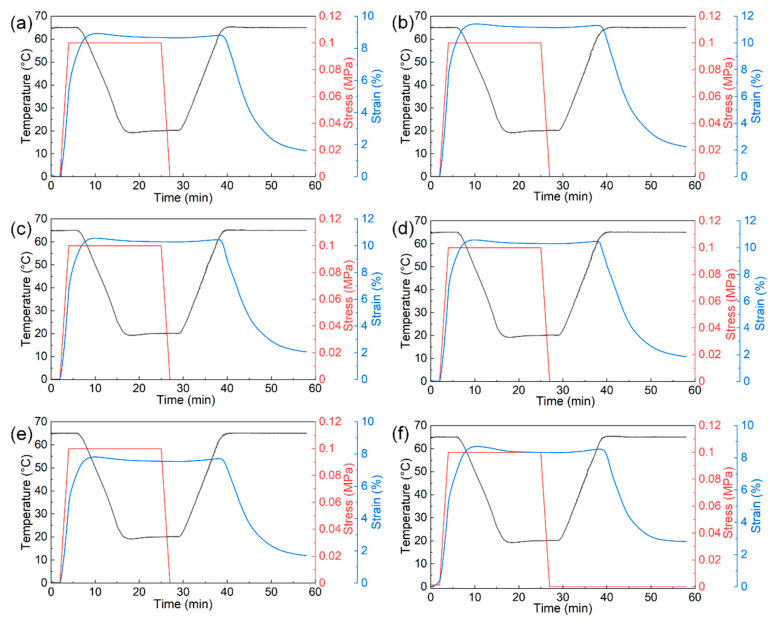
Shape memory cycle curves of (**a**) PLA/PCL-20, (**b**) PLA/PCL-40, (**c**) PLA/PCL-60, (**d**) PLA/PCL80, (**e**) PLA/PCL-100, (**f**) PLA/PCL-HP samples.

All the printed 4D samples with different infill densities exhibit good shape memory properties by DMA tests in controlled force mode, as shown in Figure 6. During the shape memory cycle, the strain of sample increases with the loading of stress in the first region. After maintained in the plateau region of fixing process, the strain is finally dropped smoothly toward to the original state in the last region of recovery process, which indicates good shape memory behaviors with a favorable fixing rate ($R_{f}$ ≥ 95%) and an acceptable recovery rate around 80% (except PLA/PCL-100). The calculated $R_{f}$ and $R_{r}$ of the printed and hot-pressed samples are summarized in Table 4. The infill density is found to have little effect on the $R_{f}$ and $R_{r}$ of the printed samples. The $R_{f}$ and $R_{r}$ values of the 100%-infilled sample decrease slightly, while the values of other samples are almost the same. The printed PLA/PCL samples show better shape memory effect compared to the hot-pressed sample, which may indicate that the printed samples with porous structures are more likely to return to their original shape during the recovery process.

**Figure 7:**
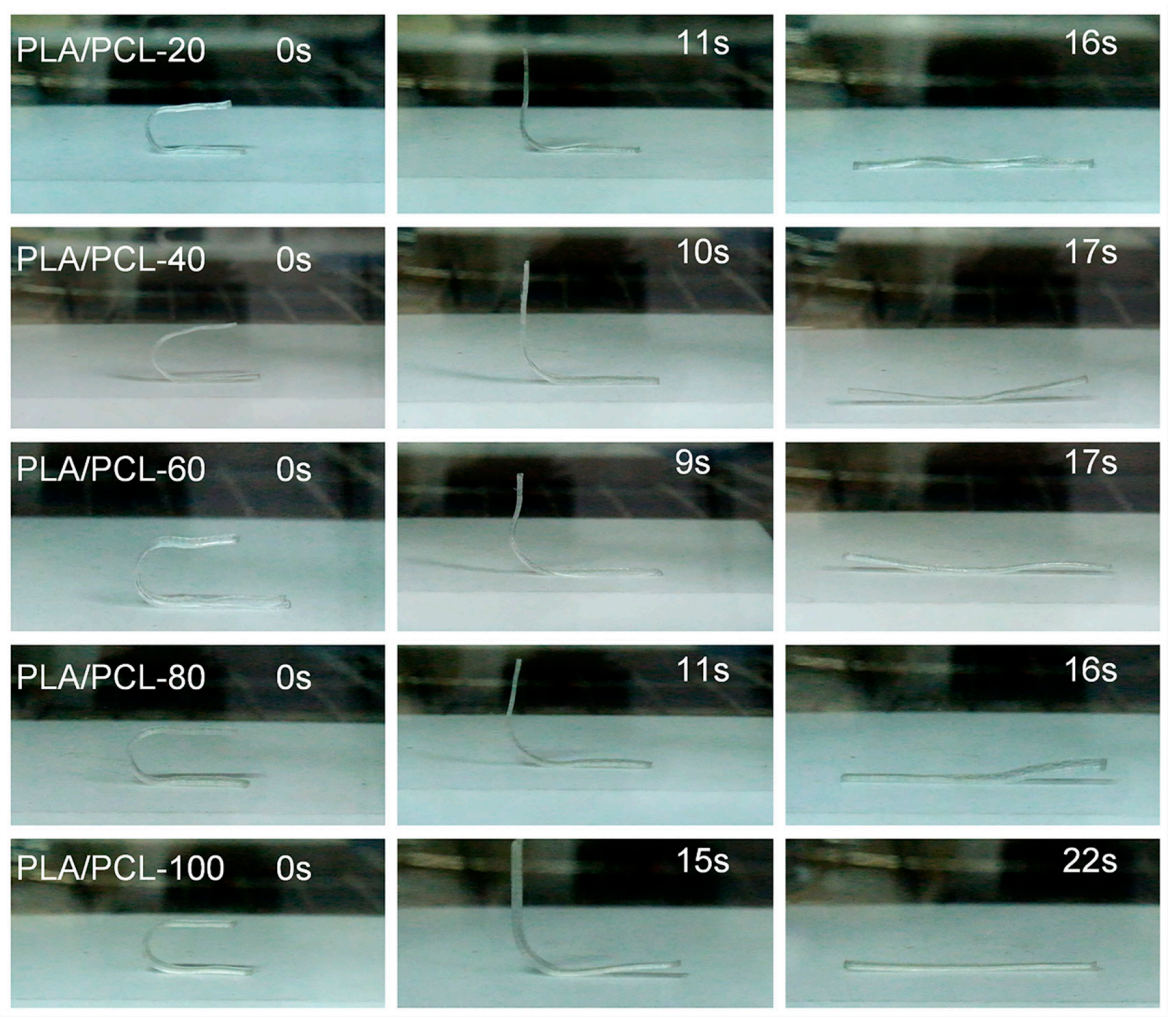
Visualization of shape recovery processes for printed 4D samples.

The visualization of shape recovery processes for printed 4D samples are shown in Figure 7. The shape memory recovery times of PLA/PCL printed samples with infill densities from 20% to 80% are about 16–17 s from heating to recover the original shape, while the recovery time of PLA/PCL-100 is a little longer to 22 s. This is attributed to that the heating of sample with 100% infilled density is more difficult, which slows down the activation of the shape memory process. It is consistent with other reference that the higher the infill density, the slower the recovery speed [60].

## 4. Conclusions

In this work, the filaments of PLA/PCL blends have been prepared by extrusion method and the PLA/PCL 4D samples with infill densities of 20–100% have been prepared by ME printing technique. The typical sea-island phase separation microstructures of printed samples are observed with PCL droplets homogeneous distributed in the PLA phase and deformation along the printing direction. The thermal properties of all the PLA/PCL samples are stable with initial decomposition temperatures above 200 °C, while two melting peaks are found around 57 °C and 168 °C, corresponding to those of PCL and PLA phase transition temperatures. The storage moduli of printed samples increase with the increase in infill density and decrease slowly in the temperature region of PCL phase transition from −40 to 60 °C. The prediction of effective moduli variation with temperature and infill densities are conducted by a two-step model and agrees well with the experimental results. Good shape memory properties with a favorable fixing rate ($R_{f}$ ≥ 95%) and an acceptable recovery rate around 80% are measured and scarcely influenced by the infill density. It is found that the higher infill density causes slower recovery speed, while the shape memory recovery times are found less than 22 s for all the printed 4D samples. The effects of temperature and infill density are proved to have different influences on the thermal, mechanical and shape memory properties of PLA/PCL blends. The influence mechanism and theoretical prediction model in this work can be used for the analysis and prediction of 4D printing materials based on PLA/PCL blends.

## Figures and Tables

**Table 1:** Printing parameters of PLA/PCL 4D samples.

Parameter	Value
Nozzle diameter	0.4 mm
Nozzle temperature	190 °C
Platform temperature	50 °C
Printing speed	30 mm s^−1^
Layer thickness	0.2
Top/Bottom layers	1
Infill density	20/40/60/80/100%
Infill pattern	Hexagon for 20–80%, Lines for 100%

**Table 2:** Empirical parameter ζ for various effective mechanical properties.

P	$P_{f}$	$P_{m}$	ζ
E_11_	E_11f_	E_m_	2(l/d)
E_22_	E_22f_	E_m_	2
G_12_	G_12f_	G_m_	1

**Table 3:** Parameters in two-step model for PLA/PCL printed samples.

Samples	ζ	φ	C	n	Confidence Bounds of n [95%]
PLA/PCL-20	3	41.01%	1	0.6681	(0.6569, 0.6794)
PLA/PCL-40	3	38.51%	1	0.6876	(0.6820, 0.6932)
PLA/PCL-60	3	30.16%	1	0.6538	(0.6376, 0.6701)
PLA/PCL-80	3	23.48%	1	0.7019	(0.6586, 0.7453)
PLA/PCL-100	3	13.47%	1	0.1150	(0.0401, 0.1900)

**Table 4:** $R_{f}$ and $R_{r}$ of ME printed and hot-pressed PLA/PCL samples.

Samples	Method	$R_{f}\;$	$R_{r}\;$
PLA/PCL-20	ME	97.20	81.84
PLA/PCL-40	ME	97.72	80.37
PLA/PCL-60	ME	97.45	80.42
PLA/PCL-80	ME	97.45	82.49
PLA/PCL-100	ME	96.58	78.18
PLA/PCL-HP	Hot-pressing	95.80	67.62

## Data Availability

Available on request.
